# Current Status of Mechanical Circulatory Support: A Systematic Review

**DOI:** 10.1155/2012/574198

**Published:** 2012-08-26

**Authors:** Kyriakos Spiliopoulos, Gregory Giamouzis, George Karayannis, Dimos Karangelis, Stelios Koutsias, Andreas Kalogeropoulos, Vasiliki Georgiopoulou, John Skoularigis, Javed Butler, Filippos Triposkiadis

**Affiliations:** ^1^Department of Thoracic and Cardiovascular Surgery, Larissa University Hospital, P.O. Box 1425, 411 10 Larissa, Greece; ^2^Department of Thoracic and Cardiovacsular Surgery, School of Medicine, University of Thessaly, Biopolis, P.O. Box 1400, 411 10 Larissa, Greece; ^3^Department of Cardiology, Larissa University Hospital, P.O. Box 1425, 411 10 Larissa, Greece; ^4^Department of Vascular Surgery, Larissa University Hospital, P.O. Box 1425, 411 10 Larissa, Greece; ^5^Cardiology Division, Emory University, Atlanta, GA 30322, USA

## Abstract

Heart failure is a major public health problem and its management requires a significant amount of health care resources. Even with administration of the best available medical treatment, the mortality associated with the disease remains high. As therapeutical strategies for heart failure have been refined, the number of patients suffering from the disease has expanded dramatically. Although heart transplantation still represents the gold standard therapeutical approach, the implantation of mechanical circulatory support devices (MCSDs) evolved to a well-established management for this disease. The limited applicability of heart transplantation caused by a shortage of donor organs and the concurrent expand of the patient population with end-stage heart failure led to a considerable utilization of MCSDs. This paper outlines the current status of mechanical circulatory support.

## 1. Introduction

The prevalence of chronic heart failure (HF) is worldwide increasing and meanwhile averages 2.5% of the normal population [1, 2]. In Europe and the United States, more than 17 million people suffer from the disease and more than 500,000 people are yearly newly diagnosed, while the chance for a 40-year-old individual to develop HF during its lifetime approximates 20% [3]. The economic impact of the disease is important, involving in the European community approximately 1% of the total public health expenditure and up to $40 billion in the USA [4, 5].

The prognosis of chronic HF remains poor despite a lot of recent advances in medical management, interventional therapies, and surgical techniques [6–8]. It is estimated that the mortality at 5 years approaches 80%, while patients receiving inotropes have 1-year survival less than 30% [9, 10]. Although heart transplantation is the treatment of choice for patients in end-stage HF non responding to medical treatment, its applicability is limited by a shortage of donor organs [11]. Data from the Eurotransplant registry shows that in 2001 a patient on the heart transplantation waiting list would be able to undergo transplantation within one year. In 2011, in contrast, there were 1,222 patients listed and only 553 transplantations were performed. After a transient peak in the number of heart transplants in the mid-1990s, the number of reported heart transplants has remained essentially stable. In the last decade, between 3,600 and 3,850 heart transplants have been registered yearly in the (ISHLT) Transplant Registry, which represents approximately 66% of the heart transplant procedures performed worldwide [12]. A growing number of heart transplant candidates require longterm support by a left ventricular assist device (LVAD) while they await cardiac transplantation. LVAD therapy has evolved into a standard therapy for patients with advanced HF, not only as a bridge to cardiac transplantation (BTT) but also as a bridge to decision (BTD), destination therapy (DT), or bridge to myocardial recovery (BTR) [13]. The growing excess of listed candidates without increase in the supply of donor hearts has more recently shifted the recipient population back to one of severe decompensation with high short-term mortality without intervention.

Historically, John Gibbon in 1953 was the first to introduce the idea of mechanically supporting the cardiopulmonary system, when he successfully used cardiopulmonary bypass for an atrial septal defect repair [14]. The first ventricular assist device was implanted in 1963 by DeBakey in a patient suffering a cardiac arrest following aortic valve replacement. The patient subsequently died on the fourth postoperative day. Nevertheless, DeBakey reported in 1966 the first successful implantation of a pneumatically driven VAD as bridge to recovery for 10 days in a patient sustained postcardiotomy shock. The patient ultimately survived to discharge [15]. Cooley reported soon thereafter, the first successful implantation of a pneumatically driven artificial heart as bridge to transplantation [16]. In 1984, DeVries and colleagues performed the first successful implantation of the Jarvik-7-100 total artificial heart [17]. Despite the first promising results, the incidence of thromboembolic and infectious complications remained high leading to a moratorium in 1991 regarding the use of the total artificial heart. However, in 1994, the achieved advances in the development of LVADs culminated in a Food and Drug Administration (FDA) approval of an LVAD as a bridge to transplantation treatment.

## 2. First-Generation Mechanical Circulatory ****Support

The first-generation LVADs were large pulsatile, positive displacement pumps with a lot of moving parts. The devices were limited to patients with a body surface area greater than 1.5 m² and were the first MCSDs initially introduced into clinical practice. The prototypes are the Novacor left ventricular assist system (LVAS, WorldHeart, Salt Lake City, Utah, USA), first implanted in a human in 1984 and used successfully as BTT in that patient, the Thoratec IVAD (implantable ventricular assist device) and the HeartMate XVE (later called HeartMate I; Thoratec Corporation, Pleasanton, Calif), which was first tested in a clinical trial in 1986 [18, 19]. The HeartMate XVE is the only long-term implantable MCSD not requiring systemic anticoagulation therapy, while the Thoratec IVAD is the only implantable MCSD approved for biventricular support.

Regarding the site of pump implantation commonly utilized pulsatile-flow MCSDs, in which the blood pump lies external to the patient are the Thoratec PVAD (paracorporeal ventricular assist device), the Berlin Heart Excor (Berlin Heart AG, Berlin, Germany) and the Toyobo LVAS (Toyobo Co Ltd, Osaka, Japan) fulfilling indications for temporary use for the BTT and BTR (Table 1). The clinical performance of MCSDs is evaluated in several studies. The landmark of those is the Randomized Evaluation of Mechanical Assistance in Treatment of Chronic Heart Failure (REMATCH) study, which evaluated the HeartMate XVE assist device compared to medical treatment in patients with end-stage heart failure [20]. This series consisted of 129 patients with heart failure of New York Heart Association (NYHA) class IV not fulfilling indications for heart transplantation. The study population was randomized to receive either a HeartMate XVE or maximum medical treatment. The 1-year survival was in the assist device group with 52% significantly higher compared to the medical treatment group, which showed a survival at 1 year of 25% and after 2 years 28% versus 8%, respectively. Major drawbacks, of the HeartMate XVE, are its large size, high device failure and infection rate of 17% and 41% respectively at 18 months of continued use [21]. A study of 280 patients after HeartMate XVE implantation performed by Lietz and colleagues confirmed the outcomes of the REMATCH study [22]. Despite an 1-year survival of 56%, the postsurgical early mortality and device failure rate at 2 years were fairly high at 27% and 73%, respectively.

Similar results regarding survival provided the nonrandomized series of Rogers et al., which evaluated the performance of the Novacor LVAS. The LVAD treatment led to improved survival compared to the medical therapy, but was associated with neurologic complications approaching a stroke risk rate of 62% [23].

The first-generation MCSDs have been supplanted by newer devices because of their high device-related complications such as infections and mechanical failure arizing from their large size and the complexity of the pump function.

## 3. Second-Generation Mechanical Circulatory Support

The second-generation MCSDs consisted of axial pumps, which utilize continuous rather than pulsatile blood flow without valves. This continuous pulseless blood flow is physiologically entirely well-tolerated and pulseless LVADs support improves neurocognitive disturbances due to severe heart failure, just as pulsatile devices do [24]. The presence of a single-moving rotor minimizes device wear and tear resulting to mechanical stability for years. Additionally, their smaller size makes second-generation devices less prone to infections and enables the implantation in patients with small body surface areas.

Second-generation VADs include the Jarvik 2000 (Jarvik Heart, New York, NY, USA), the MicroMed DeBakey VAD (MicroMed Technologies, Woodlands, Tex, USA) and the HeartMate II (Table 1). The HeartMate II represents to date the most frequently used second-generation pump worldwide [25–28].

The outcomes of second-generation LVADs compared to those of their first-generation counterparts were evaluated in a randomized trial comparing the Heartmate II and Heartmate XVE [25]. Survival at 2 years was significant higher (46% versus 11%) in the HeartMate II group, while the device-replacement rate was only 10% in the second-generation VAD group compared to that of 36% in the first generation VAD patients. Although the anticoagulation scheme in the HeartMate II group consisted of aspirin and warfarin, targeting an INR of 2.0-3.0, and only aspirin in the HeartMate XVE group, the risk of stroke and overall bleeding did not differ significantly between the two groups. However, the bleeding rate requiring surgical intervention in the second-generation VAD patient group was 30%. Interesting to mention is the observation that, although the enrolled patients in this trial were excluded from transplantation waiting lists, eventually 9 patients from the HeartMate XVE and 17 from the HeartMate II group underwent transplantation. The improved clinical performance of the second-generation LVADs resulted in a wider acceptance and use of the devices. Their major complications related to anticoagulation (bleeding and thrombosis) as presented in the first studies performed have been largely circumvented through technical modifications of the devices and improved anticoagulation regimes [28, 29]. A recently published standardized protocol dealing with this issue contributed to a better management of patients supported by these devises [30].

## 4. Third-Generation Mechanical Circulatory Support

Third-generation MCSDs provide like second-generation LVADs continuous blood flow, utilized from an axial or a centrifugal rotor. The impeller or rotor consists of a mechanism forced by hydrodynamic or electromagnetic energy, reducing in that way the moving parts and the areas of contact. The magnetic-levitation (maglev) system can be distinguished into three types: (i) external motor-driven system, (ii) direct-drive motor-driven system, and (iii) self-bearing or bearingless motor system. Through their technological advancements, third-generation MCSDs are estimated to be longer mechanically stable than their second-generation counterparts. Their smaller size approaching almost that of an AA battery enables the relative noninvasive complete intrapericardial implantation, adjacent to the heart with improved patient outcomes [31].

Third-generation MCSDS include the Levacor VAD (WorldHeart), HeartWare HVAD (HeartWare International, Inc, Framingham, Mass, USA), VentrAssist (Ventracor Ltd., Sydney, Australia, since 2010 Thoratec Corporation, Pleasanton, CA), DuraHeart (Terumo Heart Inc, Ann Arbor, Mich, USA) and the Berlin Heart Incor (Berlin Heart, Berlin, Germany, Table 1). Historically, the DuraHeart was the first third-generation device entering European clinical trials in 2004 [32]. Its performance was favorable with improved outcomes, supporting the market revolution towards extended utilization and further development of these devices. In a European multicenter study including 68 patients with advanced heart failure, who were listed for cardiac transplantation, the device was implanted as BTT. The device provided safe and reliable long-term circulatory support with survival rates at 6 and 12 months of 81 and 77%, respectively. During a mean support duration of 8 months, there was no incidence of pump mechanical failure, pump thrombosis, or hemolysis. Regarding the device related adverse events the most frequent were neurological complications (27%), right heart failure (27%) and infection, (18%) [33].

The clinical performance of the HeartWare HVAD pump (HeartWare Inc, Framingham, Mass) is evaluated in a multi-institutional trial in Europe and Australia including 23 patients. The primary end point of this bridge-to-transplant study was survival to heart transplant or survival to 180 days on the device, whichever occurred first. Actuarial survival after 6 months was 91% and 86% at the 1-year followup. The design of the HVAD pump enables a quick and less invasive implantation. The results to date demonstrate satisfactory long-term survival with excellent quality of life in this cohort [31]. Additionally, the HVAD system has been also successfully used for biventricular support [34].

The Berlin Heart Incor LVAD was evaluated in several studies, and it was associated with 1-year survival rate ranging from 53 to 63.4% and low adverse event rates, like thromboembolism ranging from 3.8 (0.1%/patient-year) to 23.2% (0.5%/patient-year) [35, 36].

In a clinical trial investigating the VentrAssist, the device reached a success rate of 82% (39.4% of patients had been successfully bridged to transplant and 42.4% of patients remained transplant eligible). The serious adverse event rates regarding infection and thromboembolism at 5 months were 0.8 and 0.12%/patient-year, respectively. Implantation resulted in improved end-organ function enhanced quality of life and reduced medication use [37].

The third-generation devices consist of smaller, potentially more reliable LVADs, which make long-term circulatory assist available to a wider range of the heart failure population, particularly those who are ineligible for transplantation or those with smaller body surface area.

## 5. MCSD Databases

The increasing use and the ongoing development of mechanical circulatory support, established the need for rigorous scientific data collection and registration. Therefore, in 2001 the International Society for Heart and Lung Transplantation (ISHLT) developed and implemented the MCSD Database. In 2006, the National Heart Lung and Blood Institute (NHLBI) grounded the Interagency Registry for Mechanically Assisted Circulatory Support (INTERMACS), a collaborative database, which collects information on MCSD implants in the USA. Data collection started on June 23, 2006, and through June 2011, more than 4,500 patients have been registered [38]. United States MCS centers designated by the Centers for Medicare and Medicaid Services (CMS) as destination therapy (DT) centers are required to enter all implants of durable devices into the INTERMACS database. With the goal of running a European registry, EUROMACS was founded on December 10, 2009 in Berlin, on the initiative of the two European centers with the largest clinical programs in the field of mechanical circulatory support—the Deutsches Herzzentrum Berlin and the Herz and Diabeteszentrum NRW Bad Oeynhausen—by 14 founding members. Additionally, the ISHLT is planing the introduction of an international MCS registry in order to collect device data from institutions worldwide.

The analysis of the INTERMACS collected data since its launch in 2006 revealed a dramatic change in the landscape of MCS support in the United States. From the year 2008, after the FDA approval of the HeartMate II continuous flow pump for BTT, there was a radical shift towards an extended use of continuous-flow pumps, being nowadays the most used devices with an implantation rate greater than 99% of adult primary LVAD implants. In addition, a gradual change has occurred during the past 5 years concerning the treatment strategies, reflected by the increased use of VAD as DT.

INTERMACS established 7 clinical profiles (Table 2) in order to facilitate the assessment of the need for MCSD therapy as well as the risk associated with MCSD implantation. This classification offers a more precise categorization of the disease severity in patients with advanced stages of HF than the traditional one provided by NYHA [39]. Regarding the disease severity of patients with MCSD-support there is a trend avoiding VAD implantation in patients in critical cardiogenic shock (INTERMACS level 1), as it was shown by the decreasing rates of critically ill patients with MCSD support from 35% to 17% in the most recent INTERMACS era. Those patients may be best served with initial temporary support until stabilization and recovery from the organ dysfunction is approached [27, 40, 41].

## 6. Patient Selection—Outcomes

Four broad indications for an MCSD are defined, with regard to the clinical intent at the time of implantation: (i) the bridge-to-transplant intent (BTT) performed on patients eligible for transplantation, while listed for a transplant; (ii) destination therapy (DT) for patients not eligible for transplantation having refractory heart failure symptoms; (iii) bridge to decision (BTD) including patients requiring MCS with the option of reevaluation of their candidacy for transplantation after improvement of clinical parameters through the MCS, (iv) bridge to myocardial recovery (BTR) applied to patients with nonischemic heart failure, with the goal to restore myocardial function targeting the explantation of the device. The decision to apply an MCSD to a patient is often difficult, thus the criteria for referral vary greatly among institutions, but nevertheless heart failure confirmed by typical signs such as pulmonary capillary wedge pressure > 20 mm Hg, cardiac index < 2.0 L/min/m², or systolic blood pressure < 80 mm Hg, despite best medical management, should be present [29].

The REMATCH trial firstly, as mentioned before, proved the marked survival advantage of MCSDs over chronic medical therapy. As surgical techniques, postoperative care and devices improved, the mortality rates subsequently decreased to a level of approximately 9% in some centers [42].

**Figure 1:**
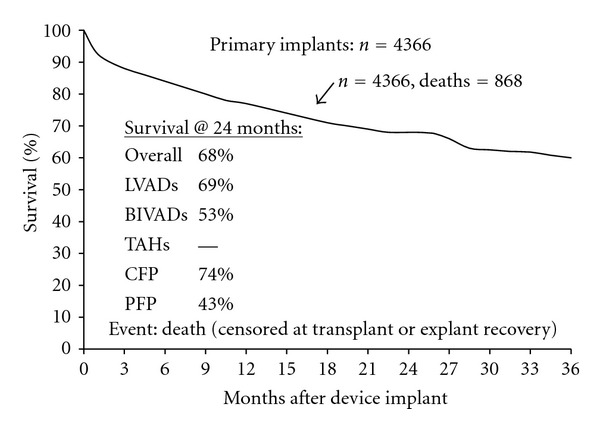
Actuarial survival (censored at transplant or explant/recovery) among 4,366 ventricular assist device implants from 06/23, 2006-06/30, 2011 and additionally stratified by device type, and pump type. (*with permission from: The Fourth INTERMACS Annual Report)* [32]. Abbreviations: INTERMACS: Interagency Registry for Mechanically Assisted Circulatory Support. LVAD: left ventricular assist device. BiVAD: biventricular assist device. TAH: total artificial heart. CFP: continuous-flow pumps. PFP: pulsatile-flow pumps.

According to the recently published annual report of INTERMACS for the entire patient population of primary MCS for the past 5 years, overall survival has progressively improved since 2006, exceeding 80% at 1 year. There is still a dramatic improvement in survival in favour of continuous-flow compared to pulsatile LVADs. Continuous-flow pumps (CFPs) had at 2 years a significant higher survival of 74% when compared to 43% of the pulsatile-flow pumps (PFPs) cohort. With regard to the treatment-intent the survival at 1 and 2 years is, as expected, less for DT, because those patients are generally not considered for transplantation as “rescue” therapy in the event of life threatening device complications (Figure 1) [38].

Apart from identifying the patient, who will benefit from MCS, similarly important is an adequate risk stratification in order to minimize the perioperative mortality of VAD implantation. In the literature, there are several series dealing with this issue, evaluating the accuracy and ability of risk stratification models in predicting early and late mortality associated with MCSD placement. In a study of Lietz et al. consisting of 280 HeartMate XVE recipients as DT, the patients were divided into high- and low-risk groups according to laboratory values and preoperative medical therapy [22, 43]. Low-risk patients showed a survival to discharge of 93.7% compared to that of 13.7% in the very high-risk group. Similarly after 1 year the low-risk group approached survival of 81.2%, while very high-risk patients presented a survival of only 10.7%. The performed mortality analysis demonstrated that comorbidities such as renal and hepatic insufficiency, right ventricle dysfunction, and poor nutritional status were independent risk factors for early mortality [8, 44, 45]. Other authors advocated the Model for End-Stage Liver Disease (MELD) score to predict perioperative and 6-month mortality after LVAD implantation [46]. Their results showed that patients with an MELD-score > 17 presented a 2.5 fold higher 6-month mortality compared to those having an MELD-score < 17. Of note is that both of the above presented studies consisted of patients with first-generation LVADs. Furthermore, these models were not derived from and have not been validated in a cohort of patients with advanced HF who are being considered for MCS. A single-center study including patients with the second-generation HearMate II assist device comparing various risk indices (Leitz- Miller, Columbia, APACHE II, INTERMACS, and Seattle Heart Failure Model (SHFM)), showed a superiority of the SHFM risk stratification model, which is a prospective validated multivariate risk-model and a tool that predicts survival of HF patients [47–51]. Summarizing, there is still need for the development of validated risk stratification models for an adequate patient selection.

According to the risk factor analysis in the recent INTERMACS annual report, during the early period (consisting of the first 3 months after implantation), the dominant mortality predictors are: clinical status of INTERMACS Level 1 (cardiogenic shock) and severe right ventricular failure sufficient to require BiVAD support (Table 4) [38]. In the constant phase (referring to the time period from 1 to 3 years after implantation), the most prominent risk factor was the presence of a first-generation pulsatile pump. The analysis evaluated subsequently subtle changes, that may occur in patient selection criteria and identified as a major change the reduction in the proportion of patients receiving continuous-flow pumps in INTERMACS Level 1 (19% versus 11%). In the most recent era, there are certain comorbid risk factors like advanced age and prior CABG-surgery, which are more prevalent. However, the fact that the expected survival (reflecting the overall risk profile in that year) has ranged from basically 81.5% to 83% for the last 3.5 years, suggests that the decrement in the strongest risk factor (INTERMACS Level 1) from 2008 to 2011 is being offset by increases in other patient comorbidities. The observation that the increase in comorbidities prevalence seems to neutralize the risk reduction effect resulting from the greater avoidance of INTERMACS Level 1 patients, while the expected 1-year survival remained relatively constant over the years (81.5% to 83%), yielded the conclusion that there is no evidence that durable device therapy is being selectively applied to lower-risk patients in the current era. 

Regarding the management of patients who need biventricular support, there is in some centers ongoing interest in the total artificial heart (TAH), seeing in the device an alternative to BiVAD support, but owing to the small number of TAH patients, there is not enough data to provide a useful evaluation of the potential positive effect of TAH.

## 7. Adverse Events

The main critical device-related adverse events include device malfunction or failure, neurological events, bleeding, infection, and right heart failure (Table 3).

### 7.1. Mechanical Failure

Mechanical device malfunctions or failures have been, particularly in the first-generation devices one of the major limiting factors concerning their long-term use. Due to their complex mechanical function pulsatile flow pumps are prone to such malfunctions. Further technological development and advancements utilized in second- and third-generation devices resulted in increased mechanical reliability and durability [29]. Current continuous-flow devices consisting of only a single, nearly moving part-the impeller- sowed in clinical trials, statistically significant lower pump replacement rates in comparison to their first and second generation counterparts [25]. Through the provided unidirectional blood flow there is no longer need for valved conduits, avoiding in that way valve deterioration. The use of hydrodynamically or magnetically levitated rotors in the newest third generation assist devices may achieve even longer mechanical reliability and durability.

### 7.2. Neurological Events

Adverse neurological events, either of ischemic or hemorrhagic origin, are a major cause of morbidity in MCSD patients. According to the INTERMACS data, primary cerebrovascular events account for 14.1% of all deaths [27]. Continuous flow devices are associated with increased thrombogenicity, which requires appropiate anticoagulation with concomitant administration of aspirin and warfarin (current INR goal: 1.5–2.5) [30]. However, in a study evaluating the HeartMate II, the incidence of neurological adverse events was comparable to that of pulsatile pumps [29, 52]. In the literature there are conflicting opinions about the adequate anticoagulation regimen that minimizes the bleeding risk and prevents thromboembolism. One advocate the “bridging” with heparin whenever a patient is subtherapeutic, while the study group of Slaughter et al. suggests that a heparin bridge might not be necessary after LVAD implantation [53]. Apart from the role of anticoagulation in the incidence of neurologic adverse events, several factors and their influence in causing strokes have been evaluated. In a recently published study including 307 consecutive patients, who underwent LVAD surgery (167 HeartMate I and 140 HeartMate II devices) at Columbia University Medical Center between November 2000 and December 2010, pre- and postoperative factors associated with neurologic complications were investigated [54]. The authors demonstrated that overall frequency of neurologic complications (NCs) including TIA after LVAD placement was 14.0% and this of ischemic/hemorrhagic cerebrovascular accident (CVA) 11.4%. The frequency of NCs was not different between patients with HeartMate I and HeartMate II devices; history of CVA and postoperative infection were independently associated with development of NCs after LVAD placement. The combination of prior CVA, preoperative sodium and albumin, postoperative sodium, hematocrit and albumin, and postoperative infection could discriminate patients who develop NCs with a discriminant probability of 76.6%. Additionally, the analysis performed for CVA patients after excluding patients with only TIA yielded similar results.

### 7.3. Bleeding

Analysis of the 2nd INTERMACS annual report demonstrated that bleeding, either at the site of implantation or in the gastrointestinal (GI) tract, was the second most frequent (16.52%/patient-month) adverse event (after infection) in MCSD patients [27]. On the other, bleeding events were infrequently fatal and accounted for only 6.7% of all deaths. 

The association of LVAD placement and bleeding has been described in several series. Strauch et al. compared GI bleeding rates among 20 HMII, 9 HeartMate XVE, and 4 VentrAssist recipients [55]. Eight patients (40%) in the HMII group developed GI bleeding, whereas no GI bleeding occurred in the other LVAD groups. 

A study of patients supported by the Novacor pulsatile LVAD—which necessitates anticoagulation—reported an incidence of nonsurgical bleeding as high as 32% [56], while a retrospective review of the European experience with the Novacor LVAD over 3 years documented a postimplantation bleeding rate of 35% (35 of 101) [57]. 

Theories that have been proposed to explain this association, assume that in continuous-flow LVADs, the impeller mechanism may cause von Willebrand's Factor (vWF) deformation, proteolysis, and an acquired deficiency of high molecular weight (HMW) vWF multimers, which predisposes to bleeding, especially in the setting of antiplatelet use [58]. Additionally, the utilized continuous-flow has led to more frequent incidence of atrioventricular fistulas in the GI tract, a finding also seen in another narrow pulse state like aortic stenosis [59]. Nevertheless, anticoagulation treatment must not be discounted as a factor in post-LVAD implantation bleeding.

It is likely that bleeding adverse events will decrease in frequency in light of the constantly evolvement of anticoagulation regimens with lower INR levels, the avoidance of heparin administration and the use of advanced methods of monitoring like thromboelastography.

In general, the adequate level of antiplatelet and anticoagulant treatment in order to avoid both thromboembolic and hemorrhagic adverse events is unknown and seems to be device specific.

### 7.4. Infection

The INTERMACS registry reported infection as the most common (17.46%/patient-month) adverse event accounting for 16.2% of all deaths, a finding which confirmed similar results from the REMATCH and HeartMate II BTT and DT trials [27]. Infection adverse events are most common within the first 3 months after placement and have a statistically significant negative influence on survival. The risk for infection after LVAD placement for long-term support is likely a multifactorial phenomenon. Newer continuous flow second- and third-generation devices are much smaller and, as the HeartMate II trials showed, associated with lower rates of pocket and driveline infections, which had been a source of morbidity and mortality. In a study performed by Martin et al., the HeartMate XVE presented an increased risk for subsequent infection when used for long-term support compared to other device types, while, the HeartMate II had a decreased risk of infection compared to the other device types used in the series [60]. These findings were explained by the smaller driveline diameter found in the HeartMate II. Previously published studies comparing clinical outcomes between other types of continuous and pulsatile LVADs have not shown any differences in terms of infectious outcomes for short- or long-term support, suggesting that the flow mechanism itself does not play a role in the risk for infection [61, 62]. There are several suggestions how to minimize the driveline infection risk. These include, among others, antimicrobial device coating or device dipping in antimicrobial/antibiofilm solution (especially the driveline) or skull fixation for the transcutaneous power lead as reported by Westaby and associates [63, 64].

Furthermore, it appears that certain subgroups of patients (e.g., patients with critical cardiogenic shock) are at highest risk for infection [65]. Several authors reported that diabetes was associated with an increased risk of death regardless of the type of VAD infection [66–68].

Improvements in device design and better patient selection strategies, particularly aiming to identify individuals with genetic susceptibility to device-related infections, may further reduce this prevalent complication and increase outcomes in patients with MCSDs [65].

### 7.5. Right Heart Failure

Although excellent survival and outcomes were documented in patients, who receive LVADs not all patients progress smoothly, due to early right heart failure (RHF) and failure to thrive (FTT), despite hemodynamic improvements. The occurrence of RHF after LVAD placement has gained attention recently, because it is associated with significantly higher perioperative mortality and morbidity rates [69, 70]. Data from the INTERMACS shows that the need for BiVAD support is associated with marked reduction in survival [38]. The actuarial survival at 3, 6 and 12 months after device implantation was for LVAD patients 90, 86, and 80% and for BiVAD recipients 70, 62 and 55% respectively. A risk factor analysis for the entire patient population of primary MCS for the past 5 years revealed patients with severe right ventricular failure sufficient to require BiVAD support as the most dominant early-mortality predictor (first 3 months, HR:3.27, *P* < 0.0001, Table 4). As soon as there are no established indications for BiVAD use, it is very important to identify patients, who are potentiall candidates for BiVAD support. Various predictors of post-LVAD RHF have been pruposed, yet few have been supported by multiple investigators [71, 72]. Fitzpatrick et al. developed a risk scoring system derived from analysis of 266 LVAD placements, including 5 clinical criteria: cardiac index ≤ 2.2 L/min*·*m², RV stroke work index ≤ 0.25 mmHg/L*·*m², serum creatinine ≥ 1.9 mg/dL, previous cardiac surgery and systolic blood pressure ≤ 96 mm Hg [73]. The subsequently constructed algorithm predicts the risk of RVAD in patients requiring LVAD therapy with >80% sensitivity and specificity [74]. Early RVAD implantation, based on the aforementioned algorithm, resulted in a significant higher survival to hospital discharge (51% versus 29%, *P* < 0.05), accompanied by higher survival at 1 year and long term.

## 8. Future Development

Second- and third-generation devices providing rotary continuous flow do not require volume compensation, but the energy transmission is still utilized percutaneously. The ideal MCSD is a fully implantable miniaturized device incorporating a transcoutaneously rechargeable battery. This is technically feasible through a transcutaneous energy transmission system (TETS). The main operation principle of the system consists of the inductive coupling of energy between an external primary and an internal subcutaneously placed secondary coil. An LVAD (LionHeart 2000 LVAD, Arrow International, Reading, Pa, USA) and a TAH (AbioCor TAH, Abiomed, Danvers, Mass, USA) are sufficiently supplied by TETS, but neither of these devices, due to other reasons not related to the TETS, is currently clinically applicable [75].

Future development of MCSD technology is being geared to the constantly changing requirements of the patients, who need or will need VAD support. As long the population of patients, who require circulatory support for end stage HF expands, future MCSDs have to be designed for long-term use, safe and less invasive implantation technique, minimizing in that way the associated morbidity. 

The refinement of the clinical classification, including patient risk profiles like those proposed by the INTERMACS study and the increasing development and use of validated risk stratification models preceded further development of sophisticated therapeutic strategies. These consist in some cases of temporary circulatory support, known as bridge to bridge therapy, in order to “prepare” the patients for permanent LVAD support. 

The Synergy Pocket Micro-Pump (CircuLite, Inc, Saddle Brook, NJ, USA) is the first miniaturized pump constructed to utilize partial circulatory support with a blood flow up to 4.25 L/min. It is implanted superficially in a “pacemaker-like” pocket through a small right thoracotomy. The device is currently undergoing clinical investigation at multiple centers in Europe with favourable initial results, aimed at achieving CE Mark [76]. The initial results of the clinical pilot study provide a proof of concept in at least a few cases, that temporary support provides important mid-term hemodynamic support in “less ill” patients. Additionally Circulite started to work on the development based on the Synergy Micro-Pump of a modified device for right heart support.

 Another minimally invasive implantable assist device is the Symphony by Abiomed (Danvers, Mass). It is the first synchronized implantable heart pump timed to an ECG, providing in that way counter pulsation therapy through a vascular graft anastomosed to the subclavian artery. Symphony was developed to treat patients with moderate HF by improving coronary perfusion and cardiac output and aiming to stimulate the LV remodeling. The device is currently under clinical investigation (Symphony: The Implantable Counter Pulsation Device (CPD) Safety and Feasibility Trial; http://clinicaltrials.gov - ID NCT01543022).

The change in the role of LVAD support as DT away from “a last option”—treatment to in some cases an “elective” therapy is under investigation in currently running studies. The one is the Risk Assessment and Comparative Effectiveness of Left Ventricular Assist Device and Medical Management in Ambulatory Heart Failure Patients (ROADMAP) trial, which is a prospective, multi-center, nonrandomized, controlled, observational study to evaluate the effectiveness of the Thoratec HeartMate II Left Ventricular Assist System (LVAS) in comparison to Optimal Medical Management (OMM). The study involves ambulatory advanced HF patients not yet dependent on intravenous inotropic support, who are typically classified as INTERMACS profiles 4–6, within the existing FDA-approved indication for D T. It will include 200 patients at up to 50 sites, including experienced HeartMate II implant centers as well as community centers that care for a large volume of advanced heart failure patients. Apart from the primary above mentioned study-objective, secondary aims of the trial are to determine the accuracy of risk prediction models of a population appropriate for HeartMate II and to establish equipoise, to determine factors related to patient and physician decisions for HeartMate II, to evaluate the frequency of cross-over to other advanced HF therapies, to compare results of early versus delayed LVAD implantation, to determine the feasibility of enrolling target population and to use information to design follow-up studies; randomized trials, or additional observational studies and registries. The trial's estimated completion date is December 2015 (http://clinicaltrials.gov-ID NCT01452802).

The second trial, the Randomized Evaluation of VAD Intervention before Inotropic Therapy (REVIVE-IT) trial, is a by the NHLBI-sponsored randomized trial of the HeartWare Ventricular Assist System (VAS) versus best medical treatment in patients with advanced HF and whose illness is not severe enough to qualify them for cardiac transplantation or permanent LVAD therapy according to current guidelines. The hypothesis of the study is that VAD therapy may improve both survival and quality of life in moderately advanced HF patients who are neither inotrope-dependent nor exercise-intolerant and have not yet developed serious complications. The pilot study will include 100 randomly assigned patients in a 1:1 ratio to the HeartWare HVAD, or optimal medical therapy and the estimated completion date is January 2016 [31].

## 9. Conclusions

As the available donor hearts for transplantation remain relatively fixed and the number of patients with end stage HF is expanding, the need for long-term circulatory support is expected to increase. Meanwhile, MCS has evolved from a last resort life-saving therapy to a well established viable alternative for thousands of HF patients. The device technology has been evolving rapidly, with both frequent advancements to the particular device types and, more recently, a dramatic shift toward the use of newer generation continuous-flow miniaturized devices. In order to determine the optimal timing when the survival benefit of MCS would be greatest by the lowest surgical risk, further prospective studies are warranted to explore on the one the safety and effectiveness of MCS in less acutely ill HF patients and to develop validated risk stratification models for adequate patient selection on the other. 

## Figures and Tables

**Table 1:** Left ventricular assist devices currently in use.

Device	Manufacturer	Type	Approval
First generation			
Novacor LVAS	World Heart	Pulsatile	CE, FDA for BTT
Thoratec IVAD-implantable ventricular assist device	Thoratec	Pulsatile	CE, FDA for BTT in 2004
Thoratec PVAD paracorporeal ventricular assist device			CE, FDA for BTT in 1995 and for post-cardiotomy recovery (open heart surgery) in 1998.
HeartMate XVE	Thoratec	Pulsatile.	CE, FDA for BTT in 2001 and DT in 2003.
Excor (paracorporeal)	Berlin Heart	Pulsatile	CE, FDA for BTT, BTR
Toyobo LVAS (paracorporeal)	Toyobo Co Ltd	Pulsatile	Approved in Japan
Second generation			
Jarvik 2000	Jarvik Heart	Continuous	FDA for BTT; CE for BTT, BTR, DT
HeartMate II	Thoratec	Continuous	CE; FDA for BTT in 2008, DT in 2010
MicroMed DeBakey VAD	MicroMed	Continuous	CE for BTT, BTR, DT; FDA for pediatric use (BTT) of the children typ in USA
Third generation			
Levacor VAD	WorldHeart	Continuous fully magnetically levitated	In February 2011 World Heart suspended enrollment in the BTT study while it awaited notification from the FDA
HVAD	HeartWare	Continuous through centrifugal blood path and hydromagnetically suspended rotor that may be placed in the pericardial space.	CE in January 2009. US BTT trial in October 2008 and US DT trial in August 2010.
VentrAssist	Ventracor	Continuous by a hydrodynamically suspended centrifugal rotor.	CE in EU and approved in Australia. Company declared bankrupt while clinical trials for FDA approvalin 2009. Intellectual property sold to thoratec.
DuraHeart	Terumo	Magnetically levitated centrifugal pump	CE; FDA trials underway
Incor	Berlin Heart	Continuous by a magnetically suspended axial flow rotor.	CE; entered clinical trials in the US in 2009.

CE: *Conformité Européenne*; European Conformity,

FDA: food and drug administration,

USA: United States of America,

EU: European Union,

BTT: bridge to transplant,

BTR: bridge to recovery,

DT: destination therapy.

**Table 2:** Interagency registry for mechanically assisted circulatory support (intermacs) levels (*with permission from* [39]).

Profile 1	Critical cardiogenic shock	Patient with life-threatening hypotension despite rapidly escalating inotropic support and critical organ hypoperfusion, often confirmed by worsening acidosis and/or lactate levels.

Profile 2	Progressive decline	Patient with declining function despite intravenous inotropic support, which may be manifest by worsening renal function, nutritional depletion, and inability to restore volume balance.

Profile 3	Stable but inotrope-dependent	Patient with stable blood pressure, organ function, nutrition and symptoms on continuous intravenous inotropic support (or a temporary circulatory support device or both), but demonstrating repeated failure to wean from support due to recurrent symptomatic hypotension or renal dysfunction.

Profile 4	Resting symptoms	Patient can be stabilized close to normal volume status but experiences daily symptoms of congestion at rest or during activities of daily living (ADL). Doses of diuretics generally fluctuate at very high levels. More intensive management and surveillance strategies should be considered, which may in some cases reveal poor compliance that would compromise outcomes with any therapy. Some patients may shuttle between Profiles 4 and 5.

Profile 5	Exertion intolerant	Comfortable at rest and with ADL but unable to engage in any other activity, living predominantly within the house. Patients are comfortable at rest without congestive symptoms, but may have underlying refractory elevated volume status, often with renal dysfunction. If underlying nutritional status and organ function are marginal, patient may be more at risk than INTERMACS Profile 4 and require definitive intervention.

Profile 6	Exertion limited	Patient without evidence of fluid overload is comfortable at rest, and with ADL and minor activities outside the home but fatigues after the first few minutes of any meaningful activity. Attribution to cardiac limitation requires careful measurement of peak oxygen consumption, in some cases with hemodynamic monitoring to confirm severity of cardiac impairment.

Profile 7	Advanced NYHA III	A placeholder for more precise specification in the future, this level includes patients who are without current or recent episodes of unstable fluid balance, living comfortably with meaningful activity limited to mild physical exertion.

**Table 3:** Adverse events rates (Events/100 patient-months) in first 12 months after implant for 1092 primary LVADs (INTERMACS: June 2006–March 2009, *with permission from: Second INTERMACS annual report*) [27].

Adverse event	Events	Rate
Device malfunction	113	1.98
Bleeding	944	16.52
Cardiac/vascular		
Right heart failure	108	1.89
Myocardial infarction	4	0.07
Cardiac arrhythmia	439	7.68
Pericardial drainage	86	1.50
Hypertensiona	132	2.31
Arterial non-CNS thrombosis	20	0.35
Venous thrombotic event	83	1.45
Hemolysis	31	0.54
Infection	998	17.46
Neurologic dysfunction	164	2.87
Renal dysfunction	142	2.48
Hepatic dysfunction	52	0.91
Respiratory failure	257	4.50
Wound dehiscence	27	0.47
Psychiatric episode	112	1.96

Total “burden”	**3712**	**64.96**

CNS: central nervous system;

INTERMACS: Interagency registry for mechanical circulatory support;

LVAD: left ventricular assist device.

**Table 4:** Risk factors for death in 4,366 primary implant patients: 06/2006–06/2011 (*with permission from: The Fourth INTERMACS Annual *Report) [32].

Risk factors	Early hazard HR	Early hazard *P *value	Constant hazard HR	Constant hazard *P *value
Age, older	1.54^a^	<0.0001	1.30^a^	0.0001
BSA, larger	1.48^b^	0.0006		
Female			1.36	0.01
History of:				
CABG	1.84	<0.0001		
Valve surgery	1.81	0.0007		
CVA	1.74	0.005		
Bilirubin, higher	1.10^c^	<0.0001		
Creatinine, higher	1.16^d^	0.01		
BUN, higher			1.08^e^	0.001
RA pressure, higher	1.21^f^	0.0004		
Ascites	1.55	0.007		
Pulmonary hypertension			1.49	0.03
Intermacs:				
Level 1	2.87	<0.0001		
Level 2	1.84	0.001	1.35	0.01
Bridge to candidacy			1.38	0.009
Destination therapy			1.38	0.009
Pulsatile-flow LVAD			3.01	<0.0001
BiVAD	3.27	<0.0001		
Concomitant surgery	1.36	0.01		

BiVAD: biventricular assist device; BSA: body surface area; BUN: blood area nitrogen; CABG: coronary artery bypass grafting; CVA: cerebral vascular accident; HR: hazard ratio; INTERMACS: Interagency Registry for Mechanically Assisted Circulatory Support;

LVAD: left ventricular assist device; RA: right atrial.

The hazard ratio denotes the increased risk: ^a^from age 70 to 80; ^b^of a 0.5-unit increase in BSA; ^c^of a 1.0-unit increase in bilirubin; ^d^of a 1.0-unit increase in creatinine; ^e^of a 10-unit increase in BUN; and ^f^of a 5.0-unit increase in RA pressure.
